# Tumor-to-tumor metastasis: a rare case of breast carcinoma metastasizing to a pheochromocytoma, and a literature review

**DOI:** 10.1186/s13000-019-0816-2

**Published:** 2019-05-20

**Authors:** Weiwei Tan, Lili Tao, Zhuping Zhou, Weihua Yin, Yaoli Chen

**Affiliations:** 1 Department of Pathology, Peking University Shenzhen Hospital, Shenzhen, Guangdong Province People’s Republic of China; 2 Department of Imaging, Nanjing Drum Tower Hospital, the Affiliated Hospital of Nanjing University, Nanjing, Jiangsu Province People’s Republic of China

**Keywords:** Tumor-to-tumor metastasis, Breast carcinoma, Pheochromocytoma

## Abstract

**Background:**

Tumor-to-tumor metastasis is a well-recognized but uncommon entity. Breast carcinoma is one of the most common metastatic donors. Breast carcinoma metastasizes commonly to adrenal glands. However, the co-existence of a metastatic lesion with an existing adrenal tumor is a rare finding.

**Case presentation:**

A 35-year-old woman was diagnosed with pheochromocytoma using computed tomography and ultrasound examinations. The tumor was surgically removed. Histological and immunohistochemical staining suggested that there were two components in the tumor: pheochromocytoma and metastatic cancer.

**Conclusion:**

This is the second published case of pheochromocytoma with tumor-to-tumor metastasis from an invasive ductal carcinoma of the breast. Furthermore, we highlight the importance of awareness of tumor-to-tumor metastasis in pathological diagnosis.

## Background

Tumor-to-tumor metastasis (TTM) is a well recognized but uncommon entity. Lung and breast carcinomas are the most common metastatic donors, while renal cell carcinoma and meningioma are the most common malignant and benign recipients, respectively [1–3]. Breast carcinoma is a malignant lesion and often metastasizes to the adrenal gland [4]. However, metastasis to an adrenal tumor is a rare phenomenon. We present a case of pheochromocytoma with TTM from a breast carcinoma. To our knowledge, this report is the second case of TTM from a breast carcinoma to a pheochromocytoma since Seitz and Schuder reported the first one in 1987 [5].

## Case presentation

**Fig. 1:**
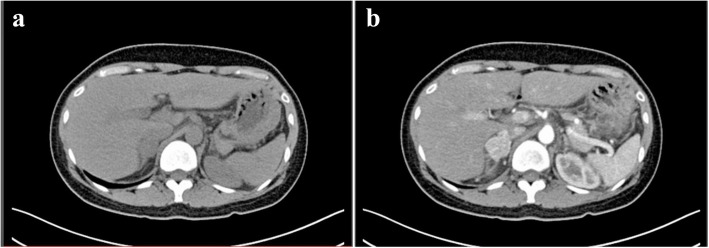
Computed tomography image showing a circular soft tissue density shadow in the right adrenal gland of the patient (**a**). The lesion in the arterial phase is markedly heterogeneous with a clear boundary after enhancement (**b**)

**Fig. 2:**
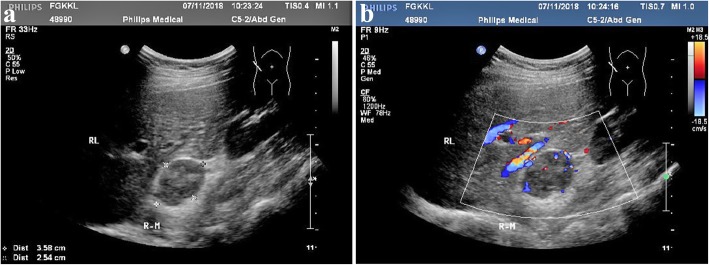
Ultrasound image showing a hypoechoic mass with a clear boundary in the right adrenal gland. The internal echo is heterogeneous (**a**). Color Doppler flow image showing a rich blood flow signal inside this mass (**b**)

One year ago, a 35-year-old woman underwent computed tomography (CT) scanning following two incidences of paroxysmal hypertension. The scan revealed a tumor above the right kidney. CT images showed a circular soft tissue density shadow in the right adrenal gland, and the lesion in the arterial phase was markedly heterogeneous with a clear boundary after enhancement (Fig. 1). A needle biopsy was performed and the pathological diagnosis was pheochromocytoma (the report was not available). The patient did not receive treatment at that time. The tumor grew slightly over the subsequent year. Then, the patient came to our hospital for treatment. Ultrasound examination again suggested pheochromocytoma (Fig. 2) and the patient underwent a tumor resection.

Analysis of the surgical specimen revealed a limited tumor measuring 3.0 × 2.5 × 2.3 cm^3^. The cut surface of the tumor had a half pinkish-grey and half whitish color. The pinkish-grey part was softer than the whitish part.

**Fig. 3:**
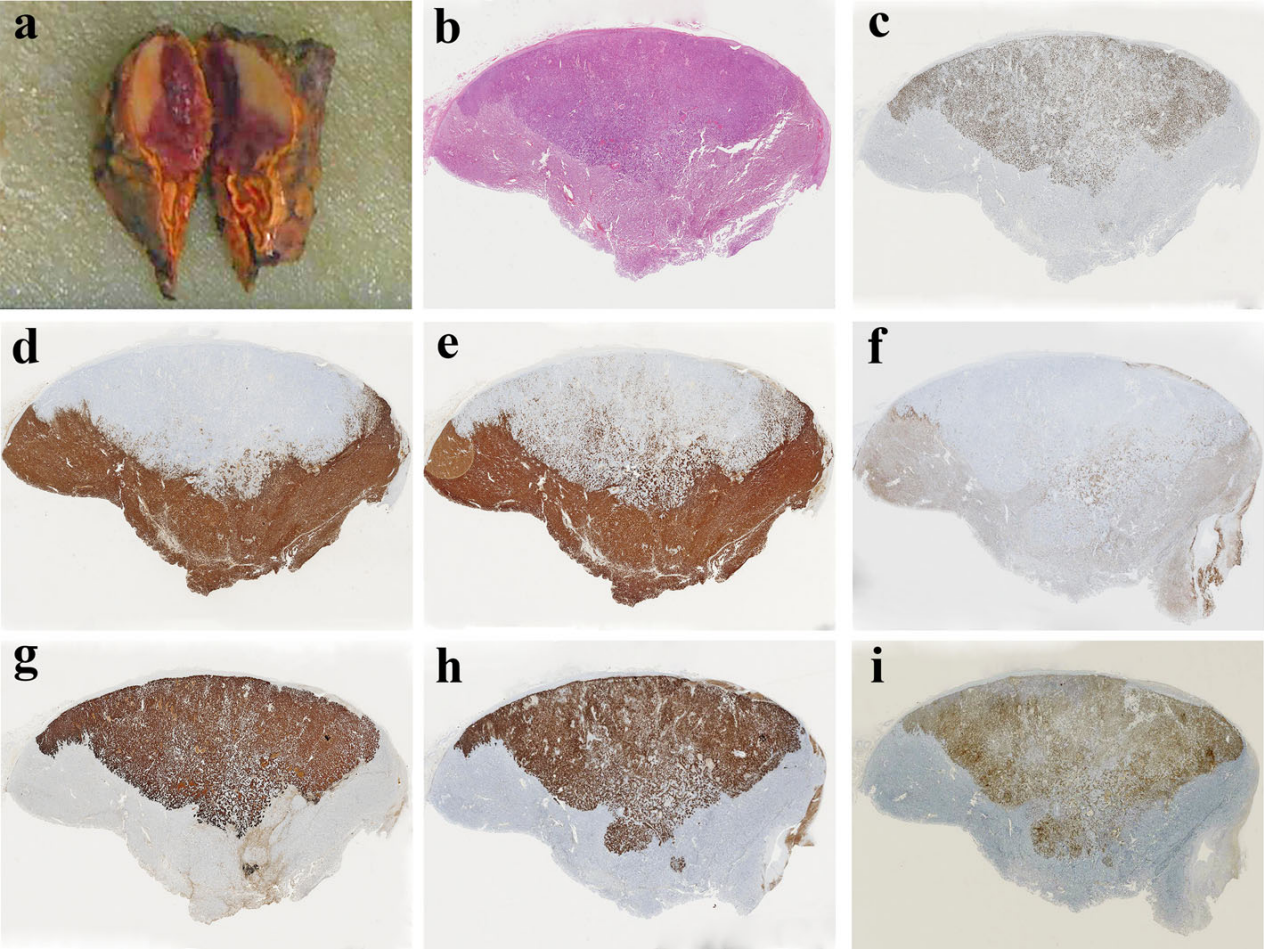
The cut surface of the tumor is divided into two parts: whitish and pinkish-grey (**a**). Histological staining with hematoxylin and eosin shows that the cells of both parts are similar (**b**). The proliferative index (Ki67) of metastatic breast cancer is significantly higher than that of pheochromocytoma (**c**). Immunohistochemistry shows that pheochromocytoma cells are strongly positive for CgA, SYN and CD56 (**d**, **e** and **f**), and metastatic breast carcinoma cells are strongly positive for CK, HER-2 and membrane positive for E-cadherin (**g**, **h** and **i**)

**Fig. 4:**
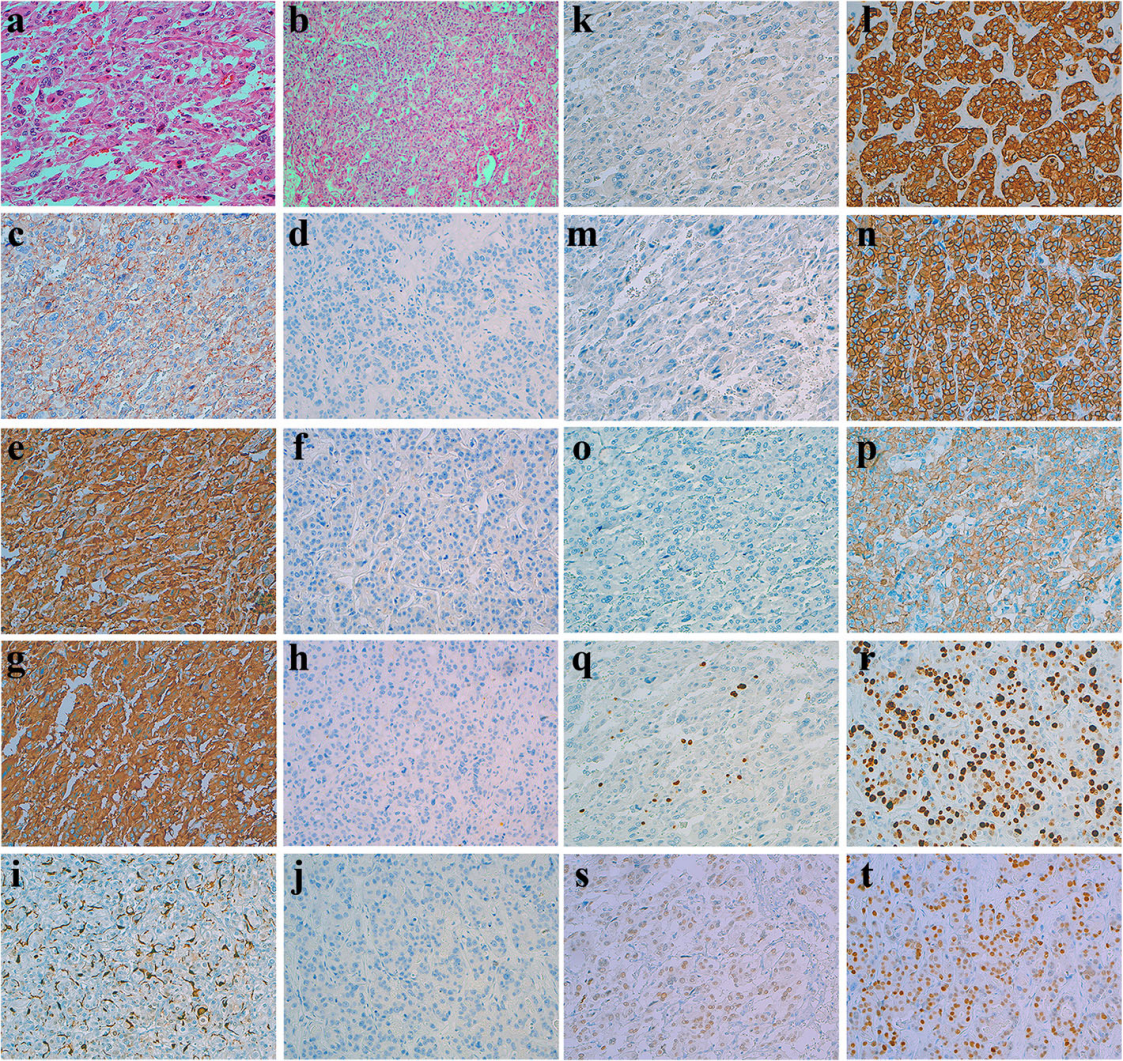
Comparison of the two tumor components in morphology and IHC staining, × 200. Hematoxylin and eosin staining of two tumor cells (**a** Pheochromocytoma, **b** Metastatic breast carcinoma). CD56 is expressed on the membrane of pheochromocytoma cells (**c** Pheochromocytoma, **d** Metastatic breast carcinoma). CgA is diffusely expressed in pheochromocytoma (**e** Pheochromocytoma, **f** Metastatic breast carcinoma). SYN is also diffusely expressed in pheochromocytoma cells (**g** Pheochromocytoma, **h** Metastatic breast carcinoma). S100 is expressed in the sustentacular cells of pheochromocytoma (**i** Pheochromocytoma, **j** Metastatic breast carcinoma). CK is stongly positive in metastatic breast cancer cells (**k** Pheochromocytoma, **l** Metastatic breast carcinoma). HER-2 is stongly positive (3+) in metastatic breast cancer cells (**m** Pheochromocytoma, **m** Metastatic breast carcinoma). E-cadherin is membrane positive in metastatic breast cancer cells (**o** Pheochromocytoma, **p** Metastatic breast carcinoma). The proliferative index (Ki67) of pheochromocytoma is not more than 10% (**q**) and that of metastatic breast cancer is about 70% (**r**). GATA-3 is weakly positive in pheochromocytoma cells (**s**) and significantly positive in metastatic breast cancer cells (**t**)

Histologically, the tumor exhibited a nest-like and trabecular growth pattern. The tumor cells were large, the cytoplasm was eosinophilic, and the nuclei were atypical. Necrosis and mitoses were obviously seen. We initially diagnosed the tumor as a pheochromocytoma. A routine immunohistochemical (IHC) assay was carried out. The results showed that part of the tumor was strongly positive for neuroendocrine markers including chromogranin A (CgA), synaptophysin (SYN) and positive for CD56, but totally negative for cytokeratin (CK). S100 was positive in the sustentacular cells, which supported the diagnosis of pheochromocytoma. Conversely, the other part of the tumor was strongly positive for CK, but negative for CgA, SYN and CD56, as well as S100. In addition, there is a significant difference in the proliferative index (Ki67) between the two parts. (Figs. 3 and 4).

Because of the particular expression pattern seen by IHC in this case, we reviewed the histological sections and found that the tumor consisted of two components. One component exhibited alveolar, trabecular, and diffuse growth patterns. The cells had a polygonal shape and were large with variably sized nuclei, and occasional bizarre giant nuclei. The other part of the tumor showed a nest and sheet growth pattern, and focal necrosis. Cytoplasm of the tumor cells was abundant and eosinophilic, and the nuclei were uniform and regular with prominent nucleoli (Fig. 4). Corresponding to the histology, the cut surface of the tumor exhibited two distinct and well-defined appearances.

Retrospectively, the patient was diagnosed with breast invasive ductal carcinoma 1.5 years ago (without mention of left or right). In addition, left retroperitoneal and thoracic vertebral metastases were confirmed. Hence, we suspected that there may be two components in this tumor: pheochromocytoma and metastatic breast cancer.

In order to verify the composition of metastatic breast cancer, we have added some known breast markers which include estrogen receptors (ER), progesterone receptors (PR), human epidermal growth factor receptor-2 (HER-2), GATA-binding protein 3 (GATA-3), Mammaglobin A, gross cystic disease fluid protein 15 (GCDFP15) and E-cadherin. The supplementary IHC assay revealed that both tumor cell types were positive for GATA-3 but negative for estrogen and progesterone receptors (ER and PR), despite the slight difference in the intensity of expression of GATA-3 in two tumors. HER-2 was strongly positive (3+), Mammaglobin A and GCDFP15 were weakly positive and E-cadherin was membrane positive in the area where cells were arranged in the nest and sheet pattern with focal necrosis, but completely negative in other areas. Representative images were displayed in Figs. 3 and 4.

## Discussion

TTM is a rare but well-established entity, although the co-existence of two or more primary tumors in an individual is relatively common. After lung, breast cancer is the second most common metastatic donor [2]. According to the literature, breast carcinoma metastases can become invasive ductal [6–10] or lobular carcinomas [11–13]. In addition, a case of mixed carcinoma involving both invasive ductal and invasive lobular carcinomas has been reported [14]. In the last two decades, TTM cases from breast carcinomas have reportedly involved tumors of the central nervous system such as meningioma [3, 15], kidney tumors like renal cell carcinoma [6, 8], and tumors of the lung, thyroid, and ovary [9, 11, 16–18] (Table 1). Among them, metastases to the former two tumor types are more common. According to some researchers, this trend may be related to the rich blood circulation in these areas [5]. The specific mechanism of TTM has not been thoroughly studied. Some experts speculate that hereditary or highly malignant tumors are more prone to TTM [1]. In the present case, the clinical stage of breast carcinoma in the patient was IV, supporting this hypothesis. However, there is no evidence of hereditary tumors.

**Table 1:** Summary of TTM cases reported in the literature from breast carcinoma

No.	Type of breast carcinoma	Primary tumor location	Age, yrs	Reference
1	Ductal carcinoma	Meningioma	63	Pham et al., 2018 [15]
2	Not mentioned	Meningioma	50	Sayegh et al., 2014 [19]
3	Invasive ductal carcinoma	Meningioma	72	Seckin et al., 2006 [20]
4	Invasive ductal carcinoma	Meningioma	69	Okada et al., 2015 [21]
5	Invasive ductal carcinoma	Meningioma	63	Lin et al., 2009 [22]
6	Invasive ductal carcinoma	Solitary renal angiomyolipoma	67	Amin et al., 2013 [10]
7	Invasive ductal carcinoma	Renal cell cancer	43	Huo et al., 2015 [8]
8	Not mentioned	Benign renal angiomyolipoma	67	Diego et al., 2013 [23]
9	Not mentioned	Renal oncocytoma	69	Bitner et al., 2017 [12]
10	Invasive ductal carcinoma	Solitary fibrous tumor	64	Velez-Cubian et al., 2016 [9]
11	Invasive ductal carcinoma	Thymic epithelial tumor	44	Moretto et al., 2013 [24]
12	Invasive ductal carcinoma	Vestibular schwannoma	57	Lua et al., 2012 [25]
13	Invasive lobular carcinoma	Follicular variant of papillary thyroid carcinoma	50	Yu et al., 2009 [11]
14	70% invasive lobular carcinoma and 30% invasive ductal carcinoma	Recurrent mysoid liposarcoma	52	Kabukcuoglu et al., 2009 [14]
15	Lobular adenocarcinoma	Granulosa cell tumor of the ovary	63	Arnould et al., 2002 [17]
16	Not mentioned	Benign ovarian fibroma	68	Perry et al., 1996 [18]
17	Lobular breast carcinoma	Superficial plexiform schwannoma	70	Gazic et al., 2011 [13]

Cases of breast carcinoma metastasizing to pheochromocytoma, and other malignant tumors to pheochromocytoma, have been reported rarely. The histological appearance of pheochromocytoma is similar to that of breast invasive ductal carcinoma, which makes the pathological diagnosis more difficult. In the present case, the cells of both tumors were large in size, similarly red-stained, arranged in nest and trabecular shapes, and contained significant atypical nuclei, and many mitotic figures. The diagnosis of pheochromocytoma is normally the first consideration, and a metastatic breast carcinoma is easily missed. In addition, it is worth noting that GATA-3, a protein commonly used to label cells of breast origin, is also expressed in pheochromocytoma cells, which may confuse the diagnosis. Thus, an IHC panel can be helpful to identify pheochromocytoma cells that are positive for CgA, SYN and CD56, but negative for CK. In contrast, cells of metastatic breast carcinoma are positive for CK, but negative for CgA, SYN and CD56. This expression pattern demonstrates the co-existence of an epithelial with a non-epithelial tumor. In addition, epithelial cells are positive for Mammaglobin A, GCDFP15, E-cadherin and strongly positive for HER-2, and supplemental information can reveal that the patient has been clinically confirmed with the occurrence of breast carcinoma metastasis at other sites (peritoneal, thoracic vertebrae, etc.), suggesting that the breast cancer is the source of the metastatic carcinoma.

## Conclusion

This rare case suggests that pathologists should be alert to the presence of TTM when two or more different histological tumors occur in the same patient or even the same mass. Especially when the morphology of the two tumors is similar, a detailed medical history and IHC assays may play very important roles in the pathological diagnosis.

## Abbreviations

CgA: Chromogranin A
CK: Cytokeratin
CT: Computed tomography
GATA-3: GATA-binding protein 3
GCDFP15: Gross cystic disease fluid protein 15
HER-2: Human epidermalgrowth factor receptor-2
IHC: Immunohistochemical
SYN: Synaptophysin
TTM: Tumor-to-tumor metastasis
